# Efficacy and Safety Profile of 25-Gauge Pars Plana Vitrectomy in Rhegmatogenous Retinal Detachment in Pakistan: A Multicenter Retrospective Study

**DOI:** 10.7759/cureus.23437

**Published:** 2022-03-24

**Authors:** Muhammad Amer Awan, Syed Zohaib Maroof Hussain, Fiza Shaheen, Mian Bilal Humayun, Nain Tara Zeb, Bushra Ayub, M. A. Rehman Siddiqui

**Affiliations:** 1 Ophthalmology, Shifa International Hospital, Islamabad, PAK; 2 Ears, Nose, and Throat, Norfolk and Norwich University Hospital, Norwich, GBR; 3 Internal Medicine, Shifa International Hospital, Islamabad, PAK; 4 Centre for Clinical Best Practices, Dean’s Office, Aga Khan University Hospital, Karachi, PAK; 5 Section of Ophthalmology, Aga Khan University, Karachi, PAK

**Keywords:** 25 gauge, proliferative vitreoretinopathy, surgical audit, pars plana vitrectomy (ppv), retinal detachment surgery

## Abstract

Introduction

This study aims to evaluate the primary anatomical success and visual outcomes of 25-gauge pars plana vitrectomy (25g PPV) in patients with rhegmatogenous retinal detachment (RRD) in Pakistan.

Design

This is a five-year retrospective, interventional cohort study conducted at tertiary care hospitals in Pakistan from October 2013 to October 2018.

Methods

This is a retrospective, interventional cohort study of 418 consecutive patients with RRD who underwent 25g PPV. All surgeries were performed by two experienced surgeons at tertiary care hospitals in Pakistan. Consecutive patients who underwent 25g PPV surgery as the treatment for RRD from October 2013 to October 2018 were included. We excluded patients who had a history of previous retinal surgery or did not complete the 4-8 weeks of primary outcome visit. We used the Statistical Package for the Social Sciences (SPSS) version 23.0 (IBM Corporation, Armonk, NY, USA) for statistical analysis. A p-value of <0.05 was considered significant.

Results

We identified 452 patients through the coding system of our hospitals who underwent 25g PPV surgery for RRD during the study period. A total of 441 patient files were reviewed for the study, of which 418 patients met the criteria for final analysis. The mean age was 49 ± 15.8 years. There was a higher number of males (n = 284, 67.9%). In our study, 186 (44.4%) patients were phakic at the time of presentation. The macula was detached in 361 (86.4%) patients. At the primary outcome visit (4-8 weeks of follow-up), the primary anatomical success rate was 89.47%. The most common cause of failure was proliferative vitreoretinopathy (PVR) (n = 20), followed by missed breaks (n = 5).

Conclusions

The surgical outcomes of RRD with 25g PPV surgery in our study were similar to the outcomes reported in the developed world. We propose a prospective multicenter national study to prospectively evaluate the risk factors for RRD surgical failure in the Pakistani population.

## Introduction

Rhegmatogenous retinal detachment (RRD) is the most common type of retinal detachment, involving one in 10,000 of the population per annum [1]. RRD causes visual deterioration due to the separation of the neurosensory retina from the underlying retinal pigment epithelium. Late presentation and proliferative vitreoretinopathy (PVR) further complicate the anatomical and visual outcomes of RRD [2].

There are different surgical methods for the treatment of RRD [3]. These include scleral buckle (SB), pars plana vitrectomy (PPV), pneumatic retinopexy, combined PPV/scleral buckle, and laser and/or cryotherapy alone for a simple, limited detachment. The trans-conjunctival sutureless 25g PPV system was first described by Fujii et al. in 2002, with the aim to reduce surgical trauma to the eye [4,5].

With developments in the vitrectomy systems, most vitreoretinal surgeons now opt for PPV for the repair of RRD [6]. The efficacy of 25g micro-incision vitrectomy surgery is well established for a variety of posterior segment pathologies [7,8].

Studies conducted in the last few years have established the safety of 25g PPV in RRD [9]. However, in previous studies, the sample size was relatively smaller, and PVR was excluded [10].

Only one study from Japan specifically showed 25g PPV outcomes in RRD complicated by PVR. The study reported the anatomical reattachment rate with a single procedure to be 78% and 93% after multiple surgeries [11]. We are unaware of any study from South Asia that reports the outcomes of 25g PPV in RRD. RRDs are more complex and behave differently in the developing world [12].

The aim of this study was to evaluate the primary anatomical success and visual outcomes in patients with RRD undergoing 25g PPV surgery in Pakistan both with and without PVR.

## Materials and methods

This is a retrospective, interventional cohort study conducted at the department of ophthalmology of Shifa International Hospital (SIH), Islamabad, Pakistan; Shahzad Eye Hospital (SEH), Karachi, Pakistan; and The Eye Centre, South City Hospital (SCH), Karachi, Pakistan. Data were collected over a period of five months (April 2020 to August 2020). All patients who underwent 25g PPV surgery as the treatment for RRD at our hospitals between October 1, 2013, and October 31, 2018, were included. Patients were identified from coding used for 25g PPV surgery. We excluded patients who had a history of previous retinal surgery. Patients who did not complete the 4-8 weeks of primary outcome visit were also excluded from the study. During the study, the patient’s privacy and confidentiality were maintained. Ethical exemption was obtained for this study from the institutional review boards of each institute (reference number 1009-284-2018).

Data were collected using a pro forma, in which information was entered from the patient’s files. Therefore, informed consent (verbal or written) was not sought. The information that was collected included patient demographics, past medical history, and risk factors (pseudophakia, trauma, RRD in the other eye, lattice degeneration, and myopia). Preoperatively, information regarding the status of lens, posterior vitreous detachment (PVD), type of breaks, number of breaks, clock hours of RD, status of the macula, presence of grade C PVR or less than grade C PVR, date in year, type of anesthesia received, and procedure performed were collected. Perioperatively, we collected information regarding laser, cryotherapy, tamponade agent, and perioperative complications. Postoperatively, the outcome measures including anatomical attachment, best-corrected visual acuity (BCVA), intraocular pressure (IOP), and postoperative complications were documented at one, two, three, and six months. Success following initial surgery was described as complete retinal reattachment at primary outcome visit (4-8 weeks). For BCVA, Snellen’s eye chart was used for recording visual acuity (VA) at one, two, three, and six months. For data analysis, VAs were converted to logarithm of the minimal angle of resolution (log-MAR) units. IOP was measured on each visit using Goldmann applanation tonometry. Posturing was advised for one week, depending on the location of retinal breaks after 4-8 hours of face-down positioning immediately postoperatively.

Surgical procedure

The surgeries were performed by two experienced surgeons (MAA and MARS) using a 25g PPV constellation vitrectomy system (Alcon Laboratories, TX, USA). To visualize the retina, a noncontact wide-angle viewing system (BIOM, Oculus Inc., Wetzlar, Germany) was used. PVD was induced in those with absent PVD with or without using triamcinolone acetonide 8 mg/0.2 mL in a 0.8 mL balanced salt solution. Membrane Blue-Dual Dye (DORC International, Zuidland, Netherlands) was inserted for one to two minutes to stain the inner limiting membrane or PVR membrane in the eyes with macular hole/PVR. Endolaser, cryotherapy, or both were applied depending on the location and number of breaks. For the tamponade effect, intraocular gas or silicone oil was injected depending on the PVR grade. Suturing of the sclerotomy sites with 6-0 Vicryl was done in cases with persistent wound leakage after the massage in patients with age < 15 years and in those with silicone oil tamponade. In every case, subconjunctival antibiotics (gentamicin) and corticosteroid (dexamethasone) were injected at the end of the procedure. Eyes with silicone oil tamponade had another surgical procedure for oil removal within 3-4 months of the first procedure. All patients had a complete ophthalmological examination at first postoperative day and were given topical antibiotics (0.5% moxifloxacin), steroids (1% prednisolone acetonide) for four weeks, and 1% cyclopentolate eye drops for two weeks. During the follow-up period, topical antiglaucoma eye drops were advised if IOP was found to be higher than 21 mmHg. Further follow-up was done after one week and then monthly.

Statistical analysis

Descriptive statistics were used to summarize the data in terms of the frequency and percentages of qualitative variables. The Statistical Package for the Social Sciences (SPSS) version 23.0 (IBM Corporation, Armonk, NY, USA) was used for data entry and statistical analysis. Quantitative variables were described as mean (± standard deviations). Visual acuity was converted from Snellen to log-MAR visual acuity. Count fingers and hand motion visual acuity were converted to log-MAR 2 and 3, respectively. The Mann-Whitney U test was applied to check the comparison between pre- and postsurgical visual acuity. The chi-square and Fisher’s exact tests were used to test for the group difference in categorical data. Visual acuity was tested before surgery and postoperatively at two and six months. A p-value of <0.05 was considered statistically significant. This is a retrospective study; therefore, a priori sample size calculation was not done.

## Results

**Figure 1:**
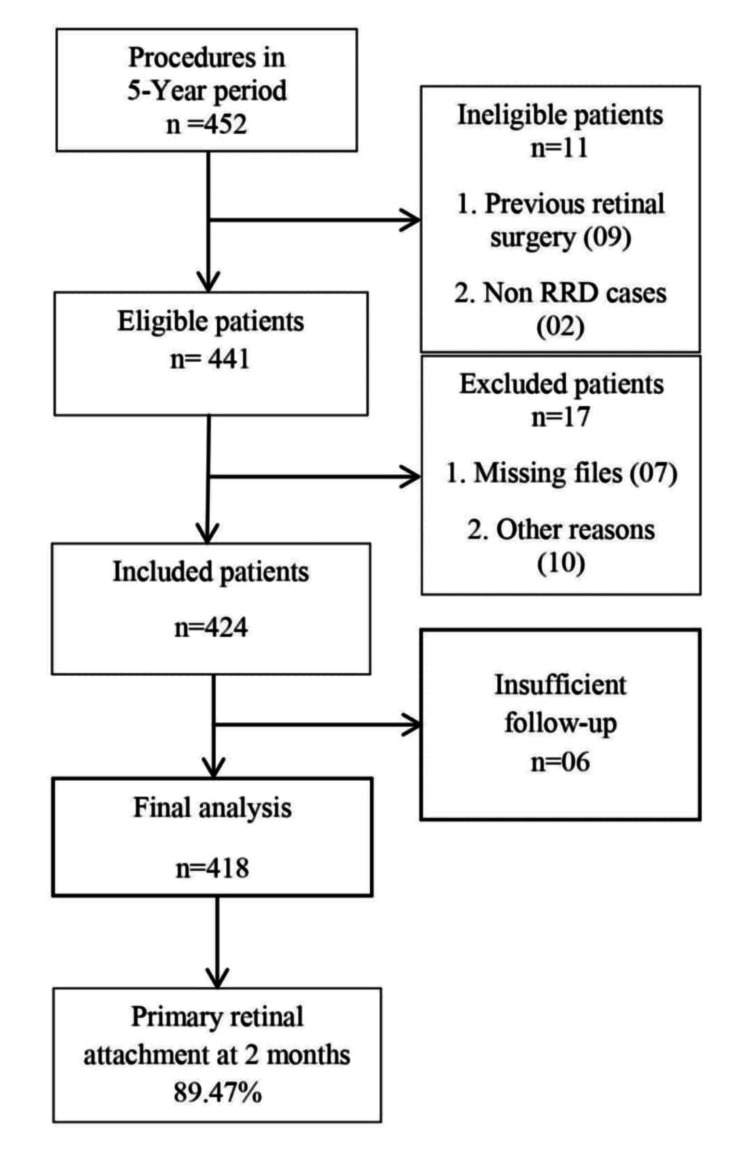
Flowchart of patient flow in the study. RRD: rhegmatogenous retinal detachment

We identified 452 patients from the hospital coding system who underwent 25g PPV for RRD during the study period, of which 11 patients were considered as ineligible either because of non-RRD cases or repeat retinal surgery. A total of 441 patient files were reviewed, of which 418 met the inclusion criteria and were included in the final analysis (Figure 1). Most surgeries were performed under local anesthesia (n = 347, 83.01%).

The proportion of males was higher (67.9%). The mean age of the patients was 49 ± 15.8 years. In our study, 186 (44.4%) patients were phakic at the time of presentation. The macula was detached in 361 (86.4%) patients. PVD was clinically present in 324 (77.5%) patients. Table 1 shows the baseline characteristics of all enrolled patients.

**Table 1:** Baseline characteristics of the enrolled patients. PVD: posterior vitreous detachment, PVR: proliferative vitreoretinopathy

Characteristics (n = 418)	Frequency	Percentage (%)
Gender		
Male	284	68
Female	134	32
Age (years) (median, IQR = 38, 60)		
5-15	17	4.1
16-25	13	3.1
26-50	167	40
51-65	165	39.6
66-80	55	13.2
Status of lens		
Phakia	186	44.4
Pseudophakia + aphakia	232	55.5
Status of the macula		
Off	361	86.4
On	57	13.6
PVD		
Present	324	77.5
Absent	94	22.4
PVR		
<PVR grade C	323	77.3
PVR grade C	95	22.7

**Figure 2:**
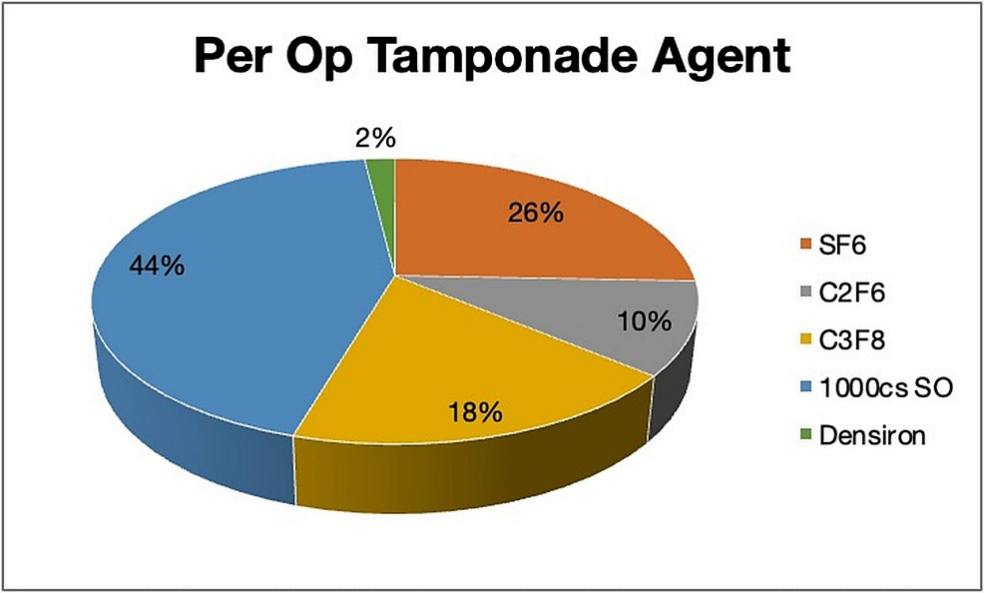
Distribution of tamponade agent used. SF6: sulfur hexafluoride, C2F6: hexafluoroethane, C3F8: octafluoropropane, SO: silicon oil

Different types of breaks were observed; the mixed type was predominant 157 (37.5%). In terms of the number of breaks, 2-5 breaks were identified in the majority of the patients (n = 221, 52.8%). Table 2 shows the intraoperative parameters of all enrolled patients. The perioperative tamponade agent used during the procedure is shown in Figure 2.

**Table 2:** Intraoperative parameters of the enrolled patients. HST: horseshoe tear, GRT: giant retinal tear

Characteristics (n = 418)	Frequency	Percentage (%)
Types of breaks		
Round holes	115	27.5
HST	95	22.7
GRT	25	5.9
Dialysis	3	0.7
Mixed	157	37.5
No tear	23	5.5
Number of breaks		
No tear identified	23	5.5
Single	106	25.4
2-5	221	52.9
6-10	42	10
>10	25	6

In our study, there was a significant improvement in the mean postoperative BCVA at six-month follow-up compared with the mean preoperative BCVA (Table 3).

**Table 3:** Comparison of pre- and postoperative visual acuity. *Median (IQR) VA in log-MAR RD: retinal detachment, N: number of patients

Time (N)	Macula-on RD*	Macula-off RD*	P-value
Preoperative (418)	0.6 (0.2-2)	2 (2-3)	<0.0001
Two months (417)	1 (0.6-2)	1 (0.7-2)	0.579
Six months (322)	0.35 (0.1-0.8)	0.70 (0.3-2)	0.002

At the primary outcome visit, the anatomical success rate was 89.47% (n = 374/418). The most common cause of failure was PVR (n = 20), followed by missed breaks (n = 5). No definitive reason for failure was identified in the remaining cases. The most common complication after vitrectomy was cataract formation/progression in 16.2% of participants, followed by a raised IOP in 12%, and ERM formation in 8% of the patients. No patient developed infectious endophthalmitis in our cohort.

When comparing the primary reattachment rate between patients with less than PVR grade C and PVR grade C, it was noted that 31/290 (10%) patients did not reattach in less than PVR grade C group (lost to follow-up = 4). While in the PVR grade C group, 13/81 (16%) failed at primary surgery (lost to follow-up = 2) (p = 0.425). It was also noted that silicone oil was used more frequently in patients with PVR grade C (p < 0.001) (Table 4). Interestingly, there was no difference between the tamponade agent used and the primary retinal reattachment rate (p = 0.306).

**Table 4:** Frequency of tamponade agent used. PVR: proliferative vitreoretinopathy, SF6: sulfur hexafluoride, C2F6: hexafluoroethane, C3F8: octafluoropropane, SO: silicon oil

	SF6	C2F6	C3F8	SO	Densiron	Total	P-value
<PVR grade C	104 (32.2%)	42 (13%)	72 (22.3%)	99 (30.7%)	6 (1.9%)	323 (100%)	<0.001
PVR grade C	3 (3.2%)	2 (2.1%)	5 (5.3%)	83 (87.4%)	2 (2.1%)	95 (100%)	

## Discussion

Our results indicate that 25g PPV is an effective surgical procedure for RRD, with or without PVR, in our study population. The primary anatomical reattachment rate of the retina in our study was 89.47%, which is consistent with an attachment rate of 80.8% (95% CI: 78.1%-83.3%) in other international studies, after one surgical procedure [4,10,13].

Following initial reservations due to instrument bending during peripheral vitreous shave, small-gauge vitrectomy is now becoming a popular method for primary RRD repair. Kunikata et al. reported a primary anatomical success rate of 95%. However, their sample size was only 84, and the PVR status was not mentioned in their study [14]. Likewise, Iwahashi et al. determined the anatomical success rate in 27 patients with RRD complicated by PVR [11]. In their study, the reattachment rate was 78% after the initial procedure and 93% after the final procedure.

To the best of our knowledge, our study is the first study from a South Asian country to report the outcomes of RRD with 25g PPV. In previously reported studies, sample sizes were small, vitrectomy was combined with scleral explants, or complicated cases with PVR C were excluded. Only one study specifically included grade C PVR RRD. That study too had a smaller sample size of 27 patients and reported a lower primary anatomical attachment rate of 78% [11].

In terms of the primary failure, the risk factors reported include more extensive detachment and preoperative PVR [15]. Multivariate regression analysis showed that preoperative PVR increased the risk of early surgical failure more than twofold. Each additional clock hour of detachment significantly increased the risk of surgical failure by approximately 12%. Delayed presentation may result in the detachment of the macula. Macula-off retinal detachment has a poorer visual outcome. It is known that patients with RRD in developing countries present late [12,16,17]. A study from Pakistan reported that 93.5% presented with macula-off RRD [16]. In addition, the nature of detachments in developing countries is complex [12].

In our study, 44 eyes had failed surgery at the primary outcome follow-up visit. One of the significant identifiable causes was PVR (n = 20). Missed or new breaks in the absence of PVR were the second most common cause (n = 5), emphasizing that a careful and thorough retinal examination with scleral indentation is mandatory at the end of any vitrectomy procedure. Over 9,000 Medicare beneficiaries in the USA were analyzed retrospectively for retinal repair at one year. It was reported that approximately 20% who underwent scleral buckle or PPV required reattachment surgery [18].

The rate of perioperative complications was significantly less in our study (<0.5%). Only a single case of an iatrogenic break was reported, further establishing the safety profile of smaller-gauge surgeries. Other studies have also reported lower iatrogenic breaks with small-gauge PPV, probably because cannula use prevents vitreous incarceration in the wound [19,20]. The use of small-gauge cannulas limits fluid currents and undesired movements of the retina, preventing inadvertent retinal incarceration in the cutter.

Raised IOP is a known complication of PPV, and the frequency is higher when SO tamponade is used [21]. In our study, increased IOP was reported in one-third of the eyes. However, it was transient and was well controlled with topical antiglaucoma drops. The second postoperative complication was a cataract that later required cataract surgery. We did not have cases of postoperative wound leak or hypotony. This has been attributed to a smaller gauge and oblique wound construction. However, in our study, we preferred suturing with a 6-0 Vicryl suture in case of persistent wound leak after a gentle massage in children <15 years of age due to the risk of involuntary rubbing and in those with silicone oil tamponade to further ensure the approximation of wound and prevention of wound leak. We found it very useful in achieving a good tamponade postoperatively.

None of our patients developed postoperative endophthalmitis, which has been documented around 0.2% previously [3]. In our view, it is directly related to wound architecture along with a meticulous draping technique and subconjunctival antibiotic at the end of surgery.

Visual outcomes depend on the status of macula preoperatively [22]. Early detection and intervention are crucial for better visual results. Williamson et al. evaluated 325 patients with macula-off RRD and found that the median final BCVA was 6/9 independently of symptom duration (recorded from day 1 to ≥21 days). Notably, they showed that surgery at any time between days 1 and 3 after symptom onset produced equivalent visual outcomes; nevertheless, surgery on days 4-6 conferred worse vision [23]. In our study, there was a significant improvement in the mean postoperative BCVA at six-month follow-up compared with the mean preoperative BCVA. BCVA at the six-month follow-up period was found to be better in the macula-on group compared with those in the macula-off group, thus reinforcing that timely RRD surgery is critical to achieving a better success rate and good vision. Our study has the highest rate of macula-off retinal detachment among the published studies of 25g PPV surgical outcome for RRD (Table 5).

**Table 5:** Studies reporting RRD outcomes with 25g vitrectomy. SB: scleral buckling, NR: not reported, PO: primary outcome

Author	Year	Country	Study design	Sample size	Macula-off (%)	PVR C included	Silicon oil (%)	Time at PO assessment (in weeks)	Success rate (%)
Mura et al. [9]	2009	Netherlands	Retrospective	131	59.5	No	3.1	12	92.4
Kunikata et al. [14]	2010	Japan	Retrospective	84	53.6	No	0	12	95.2
Iwahashi et al. [11]	2013	Japan	Retrospective	27	NR	Yes	11.1	<52	77.8
Dell’Omo et al. [24]	2013	Netherlands	Retrospective	41	58.5	No	NR	24	92.6
Susskind et al. [18]	2016	Germany	Prospective	25	72	No	NR	24	65
Rizzo et al. [4]	2017	Italy	Prospective	20	85	No	30	24	85
Romano et al. [8]	2017	Italy	Prospective	15	60	Yes	NR	24	93
Sborgia et al. [25]	2019	Italy	Prospective	46	52.1	No	NR	52	95.7
Present study	2020	Pakistan	Retrospective	418	86.4	Yes	44	8	89.4

The strengths of our study include multiple centers across Pakistan, large sample size, consecutive cases, the inclusion of complex cases with PVR grade C, vitrectomy surgery that was not associated with scleral explants, and sufficient follow-up of six months. The limitations of our study include its retrospective study design, loss to follow-up, and absence of a control group.

## Conclusions

The surgical outcomes of RRD with 25g PPV surgery in our study were similar to the outcomes reported in the developed world. These findings are interesting because the prevalence of PVR grade C was higher in our population. The findings from our study suggest that complicated retinal detachment can be successfully treated with small-gauge vitrectomy. We propose a prospective multicenter national study to prospectively evaluate the risk factors for the surgical failure of RRD in the Pakistani population.
